# Turning Waste into Value: Nanosized Natural Plant Materials of *Solanum incanum* L. and *Pterocarpus erinaceus* Poir with Promising Antimicrobial Activities

**DOI:** 10.3390/pharmaceutics8020011

**Published:** 2016-04-19

**Authors:** Sharoon Griffin, Nassifatou Koko Tittikpina, Adel Al-marby, Reem Alkhayer, Polina Denezhkin, Karolina Witek, Koffi Apeti Gbogbo, Komlan Batawila, Raphaël Emmanuel Duval, Muhammad Jawad Nasim, Nasser A. Awadh-Ali, Gilbert Kirsch, Patrick Chaimbault, Karl-Herbert Schäfer, Cornelia M. Keck, Jadwiga Handzlik, Claus Jacob

**Affiliations:** 1 Division of Bioorganic Chemistry, School of Pharmacy, Saarland University, Saarbruecken D-66123, Germany; sharoon.griffin@uni-saarland.de (S.G.); knassifatou@gmail.com (N.K.T.); aalmarby@gmail.com (A.A.); reem.alkhayer88@gmail.com (R.A.); polina.denezhkin@googlemail.com (P.D.); jawad.nasim@uni-saarland.de (M.J.N.); 2 Department of Biotechnology, University of Applied Sciences, Kaiserslautern, Zweibruecken 66482, Germany; karlherbert.schaefer@hs-kl.de; 3 Laboratoire de Botanique et Ecologie Végétale, Université de Lomé, BP 1515 Lomé, Togo; kagbogbo@gmail.com (K.A.G.); batawilakomlan@gmail.com (K.B.); 4 CNRS (Centre National de la Recherche Scientifique), SRSMC (Structure et Réactivité des Systèmes Moléculaires Complexes) UMR 7565, 1 boulevard Arago, Metz F57070, France; raphael.duval@univ-lorraine.fr (R.E.D.); gilbert.kirsch@univ-lorraine.fr (G.K.); patrick.chaimbault@univ-lorraine.fr (P.C.); 5 Université de Lorraine, SRSMC, UMR 7565, Nancy Cedex F-54001, France; 6 Department of Technology and Biotechnology of Drugs, Faculty of Pharmacy, Jagiellonian University-Medical College, ul. Medyczna 9, Cracow 30-688, Poland; karolawitek.poczta@interia.pl (K.W.); j.handzlik@uj.edu.pl (J.H.); 7 ABC Platform, Faculté de Pharmacie, Nancy Cedex F-54001, France; 8 Department of Pharmacognosy, Faculty of Clinical Pharmacy, Al Baha University, Al Baha 15791, Saudi Arabia; alinasser9678@yahoo.com; 9 Institute of Pharmaceutics and Biopharmaceutics, Philipps-Universität Marburg, Marburg 35032, Germany

**Keywords:** nanosizing, antimicrobial activity, phyto-protectant, *Pterocarpus erinaceus*, *Solanum incanum*

## Abstract

Numerous plants are known to exhibit considerable biological activities in the fields of medicine and agriculture, yet access to their active ingredients is often complicated, cumbersome and expensive. As a consequence, many plants harbouring potential drugs or green phyto-protectants go largely unnoticed, especially in poorer countries which, at the same time, are in desperate need of antimicrobial agents. As in the case of plants such as the Jericho tomato, *Solanum incanum*, and the common African tree *Pterocarpus erinaceus*, nanosizing of original plant materials may provide an interesting alternative to extensive extraction and isolation procedures. Indeed, it is straightforward to obtain considerable amounts of such common, often weed-like plants, and to mill the dried material to more or less uniform particles of microscopic and nanoscopic size. These particles exhibit activity against *Steinernema feltiae* or *Escherichia coli*, which is comparable to the ones seen for processed extracts of the same, respective plants. As *S. feltiae* is used as a model nematode indicative of possible phyto-protective uses in the agricultural arena, these findings also showcase the potential of nanosizing of crude “waste” plant materials for specific practical applications, especially—but not exclusively—in developing countries lacking a more sophisticated industrial infrastructure.

## 1. Introduction

Many plants are known to harbour biologically active ingredients, for instance against pathogenic bacteria, fungi and other microbes [1]. In order to unlock this “treasure chest” of biological activity for nutritional, pharmaceutical or agricultural uses, it is often necessary to employ a vast and expensive barrage of techniques, from extractions with organic solvents to the fractionation and isolation of the active substances. Once obtained, those compounds of interest subsequently have to be processed further to suitable forms of delivery (e.g., pills, creams, sprays, granules). Not surprisingly, most regions, especially in the developing world, lack the kind of industrial manufacturing basis to embark on such a sophisticated production process. This is rather tragic, as those regions, at the same time, are also rich in many plant species, which at least in theory may be useful in the fields of medicine and agriculture. Alternative application forms, *i.e.*, crude extracts or milled materials that exhibit adequate potential for treatment, would open up a new perspective for their use in developing countries.

**Figure 1:**
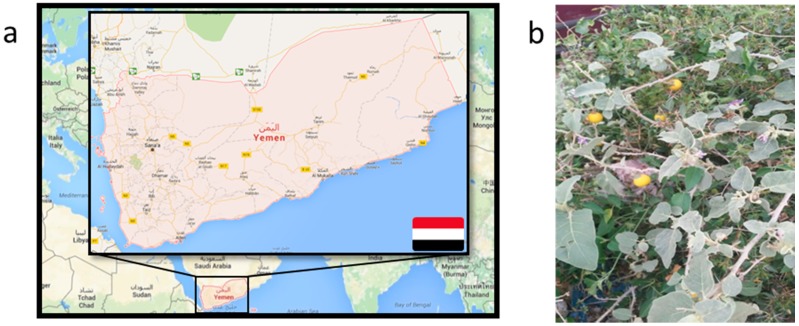

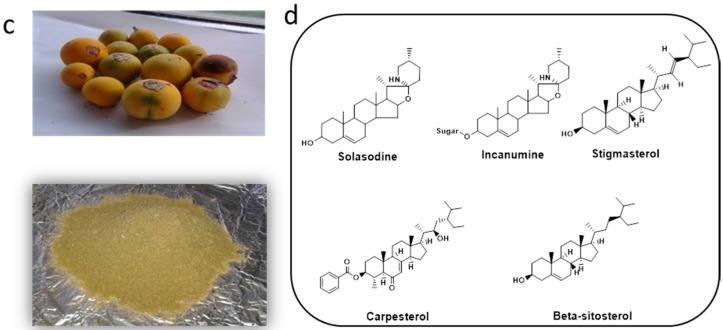
(**a**) Map of Yemen Republic indicating the area for the collection of the Jericho tomato; (**b**) This particular tomato represents a widespread agricultural weed rich in many biologically active substances. Although it is readily available across large regions of the Middle East and sub-Saharan Africa, it cannot be processed adequately in those countries and hence is of no particular use so far; (**c**) Dried fruits of *S. incanum* can be ground easily to form a green, powder-like material which in itself cannot be applied in practice. It can be processed further, for instance by extraction with organic solvents or via nanosizing; (**d**) Some of the main phytochemicals found in *S. incanum* which are considered to be responsible for its activity.

The Jericho tomato, *Solanum incanum* (in some regions also known as the bitter apple, Figure 1), is a fine example of such a plant. Growing more or less as a weed across the Arabic peninsula, the Middle East and North Africa, it is rich in many biologically highly active substances, ranging from (steroidal and glyco-)alkaloids to flavonoids and saponins [2,3]. *S. incanum* grows quickly and it has been known for over 30 years that some of its alkaloids possess an amazing antimicrobial activity against bacteria, yeasts, dermatophytes, and even some pathogens affecting agricultural produce [4]. Whilst currently of no practical use, the Jericho tomato therefore contains a vast variety of substances which may be used for a range of practical applications—if only turned into a “deliverable” form or format.

**Figure 2:**
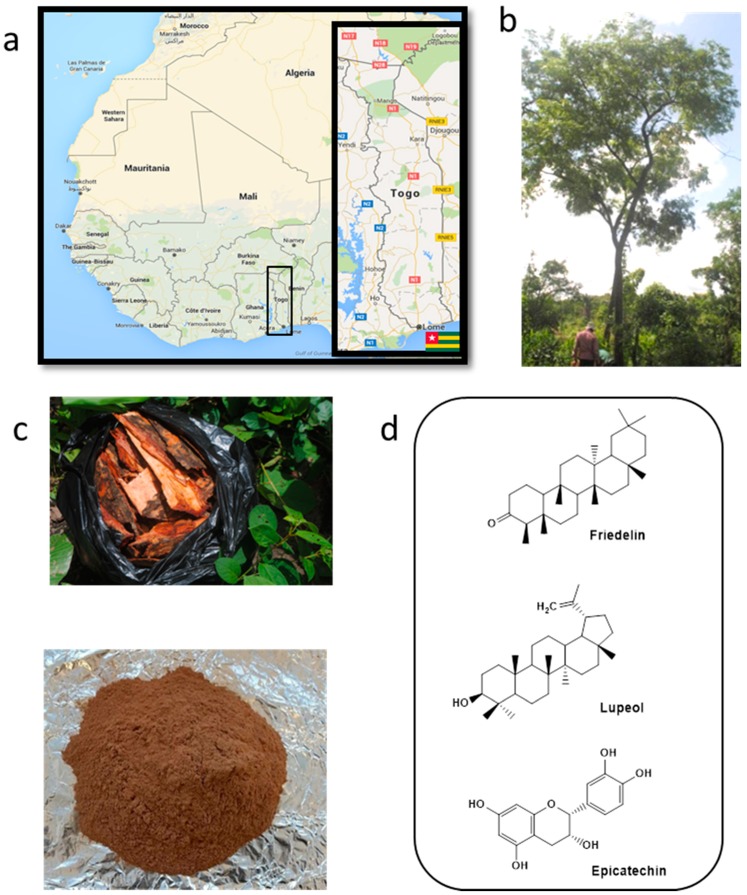
(**a**) Map showing the geographical location of Togo, home of *P. erinaceus*, in Western Africa; (**b**) *P. erinaceus* is a common, widespread and particularly rich natural source of many biologically active substances, which unfortunately cannot be used in practice due to difficult handling and low bioavailability; (**c**) Dried and powdered bark of *P. erinaceus* produced at the University of Lomé from locally collected plant material; (**d**) A brief selection of prominent phytochemicals found in *P. erinaceus* and responsible for its various biological activities.

Similarly, *Pterocarpus erinaceus*, sometimes also known as “barwood” or “vène”, represents a very common and widely grown tree native to the Sahelian region of West Africa, which is known to possess a range of potential medical uses [5] (Figure 2). The tree is widespread in the savannah areas that stretch from Senegal and Gambia to Chad and the Central African Republic [6]. The Tchamba tribe in Togo (which calls the tree “Boutô”) employs its bark to treat candidiasis and various other infections [7,8]. In contrast, the Kotokoli tribe in the same country, referring to the tree as “Tem”, uses powders obtained from its roots against typhoid fever and bark decoction against haemorrhoids [9]. Unfortunately, it is difficult for such tribes to unlock any biological or medical activities in practice, as this would require sophisticated technology. Eventually, *P. erinaceus*, like *S. incanum*, is therefore still of little advanced practical applications and is used primarily as firewood.

Faced with the problem of readily available, considerable amounts of plant material on the one side and the lack of adequate means to render these materials into a useful form on the other, we have therefore turned out attention to nanosizing little-processed, crude plant materials (please note that the expression “nanosizing” is employed here to describe the arsenal of milling and homogenization methods employed and does not imply that the materials obtained by these methods necessarily also consist of particles with diameters in the nanometer range). Here, we have posed the question if simple milling of locally readily available and otherwise useless material (due to lack of solubility and hence low bioavailability) could unlock the biological activity in a “useful” manner.

In order to achieve this goal, we have focussed on adequate, yet also readily available and economical (*i.e.*, cheaper) methods. After the initial step, *i.e.*, simple milling, a technique with a sufficiently high diminution efficacy has been selected, which is capable of destroying hard plant material and elastic plant fibres at the same time. Here, wet milling is the method of choice if very fine particle sizes are required [10]. Among the different processes for wet milling available today, *i.e.*, milling with colloid mills, bead milling and high pressure homogenization (HPH), HPH is the method of choice for the samples discussed here: the high energy input during the homogenization process enables an efficient diminution of the material within a short period of time [11]. HPH is also a well-known and hence well-established, straightforward and safe technique which is frequently applied in practice, not only in the pharmaceutical industry, but also in the field of cosmetics and food production. In addition, large scale production is possible [12]. It should also be noted that the basic HPH equipment is accepted by regulatory authorities, is of relatively low cost (around €10 k) and available worldwide.

## 2. Materials and Methods

### 2.1. Materials

In order to investigate the possibility of turning waste into value, we have chosen the two above-mentioned plants, *i.e.*, *S. incanum* and *P. erinaceus*, as they represent two common plants native to larger yet different regions of the developing world, and here two regions with a particular need of effective medicines, such as antibiotics, and antimicrobial agents for various agricultural uses, for instance as phyto-protectants.

The weed *S. incanum* was harvested by the family of Adel Al-marby on the 25 September 2015 in the Wasab District, Yemen Republic (GPS coordinates: 14°20′4″ North, 43°48′41″ East) (Figure 1). This plant has then been identified taxonomically at the Department of Botany, Faculty of Science, Aden University, Yemen, with a voucher number CP-131. Various parts of the plant, including its fruits, were subsequently dried and milled with a coffee grinder to yield the raw material shown in Figure 1c, which was afterwards used for further investigations (see below).

*P. erinaceus*, an abundant non-endangered tree, was collected in Togo in the central part of the country (GPS coordinates: 09°11′689″ North, 001°15′942″ East) on the 19 June 2014 after a formal identification by a botanist (Figure 2). Voucher specimens have been deposited at the Herbarium of the University of Lomé, Togo, with a voucher number TOGO-15077. The fresh plant material was brought to the Laboratory of Botany and Plants Ecology (University of Lomé, Togo, BP: 1515) where it was dried at 25 °C. After drying, the relevant material was ground to yield the raw material, as shown for instance in Figure 2c. These raw powders were sealed and subsequently used for further scientific investigations.

### 2.2. Nanosizing of Dried Fruit of S. incanum and Bark of P. erinaceus

Nanosizing of the dry and locally pre-processed powders obtained from Yemen and Togo (Figure 1c and Figure 2c) was performed by a combination of rotor-stator high speed stirring (HSS) and subsequent high pressure homogenization (HPH) in the presence of the natural surfactant Plantacare. The latter is a plant derived, food-grade uncharged tenside commonly used to stabilize particles destined for medical or agricultural applications. Particle size analysis was performed using Photon Correlation Spectroscopy (PCS), Laser Diffraction (LD) and light microscopy.

In the first step, the powdered, crude plant material was subjected to dry milling using a FastPrep 24 Instrument (MP Biomedicals, Solon, OH, USA). Precellys Kits (Bertin Technologies, Montigny-le-Bretonneux, France) were used as a source of ceramic beads for dry milling experiments (metallic beads were avoided as they may contaminate the sample with biologically active metal ions). After initial dry milling, and for the purpose of stabilization (*i.e.*, avoidance of aggregation), the material was suspended in 1% Plantacare^®^ 2000 UP (alkyl-polyglycoside, BASF, Ludwigshafen, Germany) in distilled water to yield 1% macro-suspensions of finely milled plant materials. Please note that Plantacare is a detergent and that water but no organic solvents were used at this step.

Subsequent pre-homogenization of these macro-suspensions was performed using a MICCRA D-9 Homogenizer–Disperser (MICCRA GmbH, Müllheim, Germany). This homogenization procedure was followed by further homogenization employing an APV Gaulin LAB 40 (APV GmbH, Mainz, Germany) High Pressure Homogenizer. The initial homogenization included three cycles at 200, 500 and 1000 bar pressure, respectively, whereas final homogenization was achieved through ten consecutive cycles at 1500 bar pressure.

In order to assess the general quality and properties of the homogenized samples, three different analytic techniques were used during the various stages of milling and homogenization, namely; LD, PCS and light microscopy. LD measurements were performed on a Mastersizer 2000, PCS measurements on a Zetasizer Nano ZS (both from Malvern Instruments, England, UK). The shape and size of the particles was assessed further by light microscopy, employing a Leica DM 1000 LED microscope (Leica Microsystems, Wetzlar, Germany). Microscopy also provided basic information regarding the homogeneity of the samples.

### 2.3. Extraction Methods

Nanosizing was paralleled by more traditional extraction methods. For this purpose, 10 g of the powdered fruits of *S. incanum* (Figure 1c) were extracted with methanol (4 × 100 mL) at room temperature and under constant shaking for 24 h. Thereafter, the extracts were combined and filtered, the filtrate was collected and the solvent evaporated *in vacuo* at 40 °C to yield the crude dry (methanolic) extract, which was stored at 4 °C until further use. The yield was calculated in percentage.

The extract of *P. erinaceus* bark was obtained by maceration of the powder of the crude plant material (Figure 2c) in a 1:1 mixture of methanol and dichloromethane for 48 h. The extract was subsequently filtered and the solvent evaporated to yield the crude extract, which was stored at 4 °C until further use. One should note that these extraction procedures—in contrast to milling and homogenization—necessarily had to involve one or more organic solvents, and that methanol rather than ethanol was chosen as the latter is often rejected for cultural or religious reasons.

### 2.4. Biological Activity Assays Involving Steinernema feltiae, Escherichia coli and Saccharomyces cerevisiae

The nanosized and stabilized particles were suspended in distilled water and then tested against three model organisms, namely the Gram-negative bacterium *Escherichia coli*, the yeast *Saccharomyces cerevisiae* and the agricultural nematode *Steinernema feltiae* [13]. Nematicidal assays were performed in the morning and antimicrobial assays in the early afternoon. Relevant experimental conditions and procedures for individual assays, e.g., data collection and evaluation, and statistical analysis have been reported by us and others already and can be found in the relevant literature [14,15,16,17].

## 3. Results

In essence, the results obtained as part of this study support the notion that it is possible to employ nanosizing as a one-step method to render crude plant materials otherwise of little or limited use into preparations with substantial biological activities. Whilst the nanosized material of *S. incanum* was particularly effective against the model agricultural nematode *S. feltiae*, the nanosized bark of *P. erinaceus* showed an interesting activity against *E. coli*. In both cases, the quality of the particles obtained was acceptable, yet could probably be enhanced further. Also, although the activity of either nanosized material was somewhat lower when compared to the respective refined extracts of those plants, the sheer ease of preparation offset the various disadvantages of those initial preparations.

### 3.1. Homogenized Particles of S. incanum

**Figure 3:**
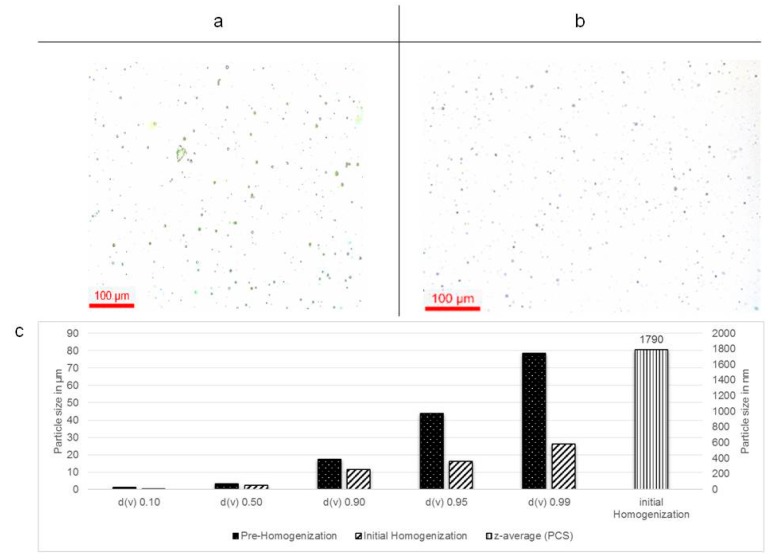
(**a**) Microscopic examination of the pre-homogenized sample of the fruit of *S. incanum* (200-fold magnification); (**b**) The same sample after initial HPH up to 1000 bar pressure; (**c**) Characterization of the samples of *S. incanum* at different stages of homogenization. Here, initial homogenization was sufficient to generate particles with diameters below 2 µm (see (**b**)), whereas further HPH at 1500 bar resulted in samples prone to aggregation (diameters above 2 µm, not shown).

This material of crudely milled *S. incanum* (Figure 1c) contains large particles of varying shapes and sizes. It is not soluble in water and cannot be used for any biological studies or applications. HSS reduced the size of the particles to about 10% of the original size and subsequent HPH led to a further break-up of the particles (Figure 3). Interestingly, an attempt to further reduce the size of the particles and/or plant cells by employing a homogenization at 1500 bar pressure did not result in a better quality of sample as this approach caused a slight agglomeration of the particles. For this reason, the samples obtained after the initial HPH cycles (*i.e.*, at “just” 1000 bar pressure), were selected for subsequent investigations in biological test systems as they encompassed the optimal, *i.e.*, smallest achievable particle sizes. These particles were more or less round in shape, and their size varied between 2 and 3 µm (Figure 3c). Eventually, the micro-particles of *S. incanum* seemed to release fibres, proteins and sugars, and hence, their physical stability was somewhat impaired and some aggregation could be observed [18]. Still, the particles, stabilized by the natural surfactant Plantacare (Figure 3a,b), formed a clear suspension in water which—in stark contrast to the original sample shown in Figure 1c—could be subsequently employed for biological tests.

### 3.2. Homogenized Particles of P. erinaceus

**Figure 4:**
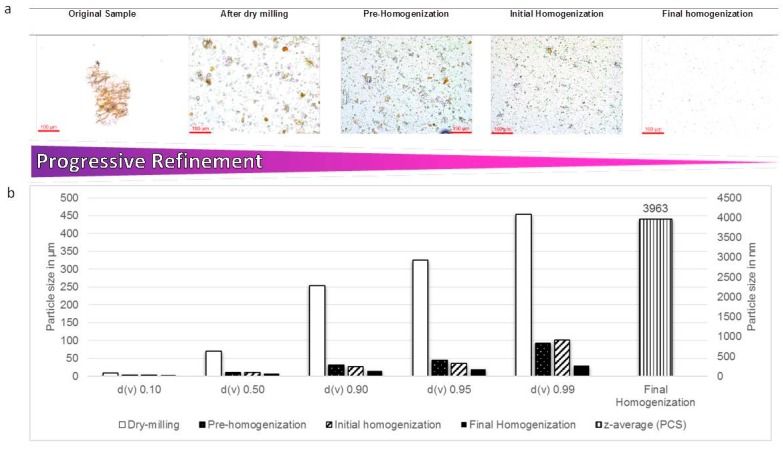
(**a**) Optical microscopy of sequentially processed bark of *P. erinaceus*, from the crude plant material ground down with a simple coffee grinder at the University of Lomé in Togo on the left to initially and finally homogenized materials on the right (200-fold magnification); (**b**) LD and PCS analysis of the different samples confirming a progressive refinement of the particles to a final average particle size of just below 4 µm achieved by HSS and subsequent HPH.

Compared to the dried fruit of *S. incanum*, the bark of *P. erinaceus* was more “hardy” and found to be more amenable for milling and subsequent homogenization. For that reason, it was also more amenable for plant specimen-specific optimization and accompanying investigations (Figure 4). After each cycle of dry-milling, the sample was therefore examined by visual inspection to ensure that it was uniformly milled down. As for *S. incanum*, the resulting particles were suspended in Plantacare for stabilization.

Pre-homogenization, which was performed at different rotor-stator speeds ranging from 11,000 up to 39,000 rpm, reduced the average diameter of the particles to around 100 μm, as confirmed by LD measurements. This initial procedure subsequently led to better results via HPH (at up to 1000 bar). After HPH, light microscopy indicated the presence of more or less round shaped particles with diameters in the one to ten micrometer range (Figure 4a). This was confirmed by PCS analysis, which estimated the diameters of the particles as just below 4 µm (Figure 4b). As for *S. incanum*, the results obtained by PCS analysis also argued against further HPH (e.g., more than ten cycles at 1500 bar), as this may lead to subsequent agglomeration. It seems that HPH can only be performed up to a certain extent depending on the properties of the individual plant material under investigation. In any case, the particle suspensions obtained by HPH were clear in appearance and quite stable at room temperature. Still, they were stored in a cool environment to reduce the possibility of long(er)-term agglomeration (see Materials and Methods).

### 3.3. Biological Activity of Processed Samples and Respective Extracts against S. feltiae

**Figure 5:**
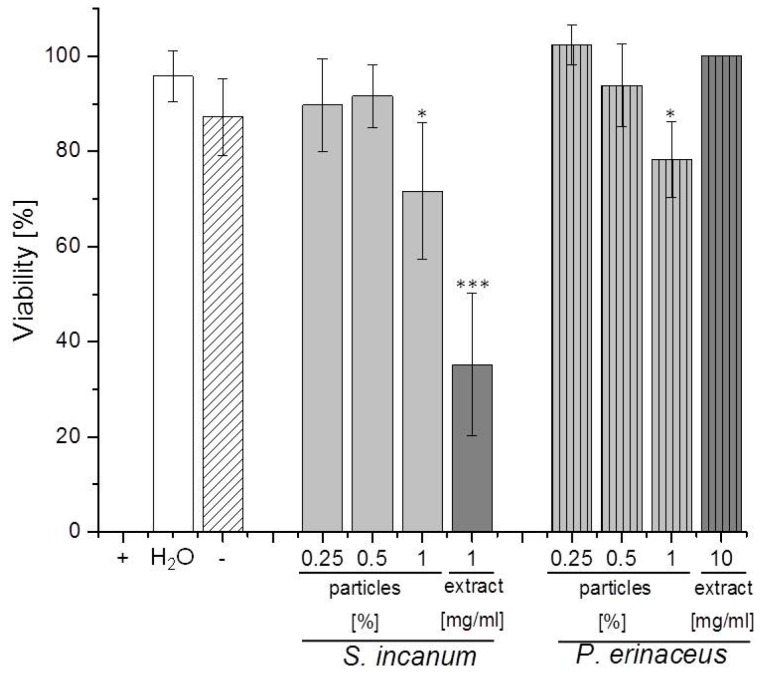
Activity of particle suspensions and methanolic extracts of *S. incanum* and *P. erinaceus* against the nematode *S. feltiae*. Experimental details are provided in the text. Negative controls (H_2_O) and Plantacare 1% (−) and a positive control (+) of 70% ethanol were used. Experiments were performed in triplicate and on at least two different occasions. Statistical significances were calculated using one-way ANOVA (OriginPlus). * *p* < 0.05, *** *p* < 0.005.

When tested for activity against any of the three representative target organisms, the particle suspension of *S. incanum* fruits, (up to a concentration of 1%) was inactive against bacteria and yeast, yet exhibited a concentration-dependent and ultimately statistically highly significant activity against the nematode *S. feltiae* (Figure 5). Here, a 1% particle suspension of *S. incanum* reduced the viability of the nematode to around 75%. At the same time, the negative controls, which included distilled water and a 1% Plantacare solution, showed no significant activity, whilst the processed methanolic extract of *S. incanum*, which was employed as a “conventional” benchmark control, was also active, reducing viability to less than 40% when used at a concentration of 1 mg·mL^−1^. Whilst it is *a priori* difficult to compare the particle suspension with the extract solution due to major differences in composition, consistency and concentration, it seems that both preparations under the experimental conditions used show an acceptable activity against *S. feltiae*.

Interestingly, a suspension of nanosized bark of *P. erinaceus* also exhibited a concentration-dependent activity against *S. feltiae*, reducing the viability of the nematode significantly, to less than 80%, when employed at a concentration of 1%. In contrast, the bark extract of *P. erinacues* did not affect the viability of *S. feltiae* (Figure 5).

### 3.4. Biological Activity of Processed Samples and Extracts of P. erinaceus against E. coli

**Figure 6:**
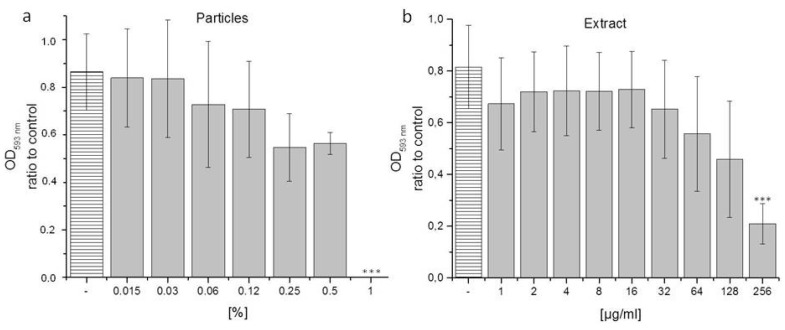
Activity of homgenized particles (**a**) and methanolic extracts (**b**) of *P. erinaceus* bark against the Gram-negative bacterium *E. coli*. Experimental details are provided in the text. LB broth (−) and Plantacare (not active, not shown) were used a negative controls. All experiments were performed in triplicate and on at least two different occasions. Statistical significances were calculated using one-way ANOVA (Origin Plus). *** *p* < 0.005.

Whilst it appears that the particles of *S. incanum*—when compared to the ones of *P. erinaceus*—are more active against *S. feltiae*, the latter display an interesting activity against *E. coli* (Figure 6). Unlike the particles of the Jericho tomato, which are inactive against *E. coli*, the particles as well as the extracts of the *P. erinaceus* reduce the survival of this Gram-negative bacterium in a concentration-dependent and statistically significant manner (*E. coli* was chosen here as intestinal and generally fairly resistant bacterial target). At a particle concentration of 1%, for instance, the bacterial count is reduced to zero when compared to the negative control (water), whilst the stabilizer Plantacare at those concentrations exhibited no significant impact on bacterial growth (data not shown). This finding is in agreement with the pronounced activity of the processed methanolic extract of the bark of *P. erinaceus*, which also reduced bacterial growth from an OD_593_ of 0.8 in the control to just 0.2 (*i.e.*, to around 25% of the original growth), when employed at a concentration of 256 µg·mL^−1^. As in the case of *S. incanum* and its activity against *S. feltiae*, the particle preparations on the one side, and the dissolved extracts on the other, are not directly comparable for several reasons. Still, it appears that processing the bark of *P. erinaceus* to small particles provides a viable method to “unlock” the biological activity of this material and hence to render it suitable to reduce the viability of certain bacteria, such as *E. coli*.

## 4. Discussion

Considered together, the results obtained with the processed, homogenized particles of *S. incanum* and *P. erinaceus* support the use of such crude materials against pathogenic organisms, such as bacteria and agriculturally relevant nematodes related to *S. feltiae* (*S. feltiae* itself is a model but not a target). On the other hand, the results also point towards possible limitations of the method and scope for further improvement, particularly in the context of particle quality and stability, aspects of release and level of activity.

The nanosizing process, in particular, deserves further attention. Whilst a combination of HSS and subsequent HPH seems to provide a simple, straight-forward and comparably rapid method to produce samples for initial biological testing, it is also apparent that there is still considerable room for further improvement. Whilst the shapes and sizes of the particles obtained by these procedures in both cases, *i.e.*, for the fruits of *S. incanum* and bark of *P. erinaceus*, no doubt are adequate for biological tests, it would be desirable to achieve particles of more uniform shapes and smaller sizes, and particularly of higher stability for practical applications in the future. Here, more refined methods, such as more appropriate stabilizers or more effective nanonization methods, e.g., ART Crystal Technology, may be considered, bearing in mind that one of the prime aims of this study has been the investigation of *simple* methods which explicitly may be applied locally in developing countries [19]. Hence future studies may have to consider a balance between economical, straight-forward methods on the one side and good quality and stable particles on the other.

Similarly, our study should also be seen as preliminary when considering issues of why such particles are active. Such considerations almost certainly will raise questions related to nanosafety and/or the release of active substances from those particles. Here, we still lack information regarding the release of biologically active compounds from them. Still, one may speculate that the particles employed serve as natural delivery systems for toxic agents such as alkaloids (see Figure 1 and Figure 2), whilst other effects on cells and organisms, such as interactions with membranes or the entire blockage of pores (in nematodes) would also have to be to be considered.

**Figure 7:**
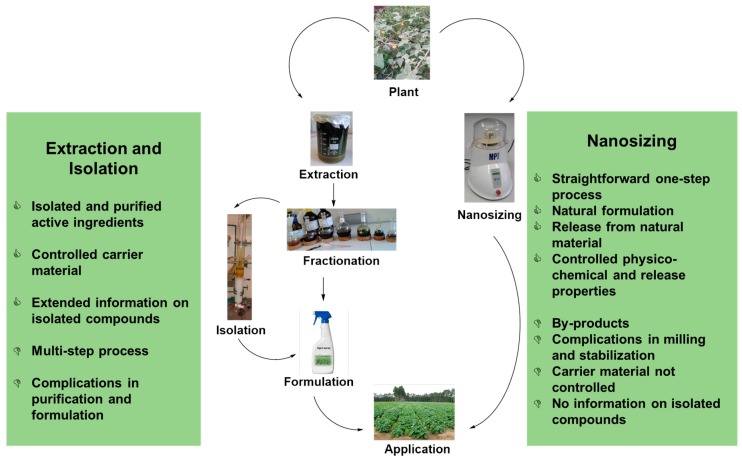
Schematic comparison of the more conventional methods to unlock the biological potential of natural materials via extraction, isolation, refinement and formulation on the left *versus* nanosizing of the crude material on the right (nanosizing may contain several steps, yet the methods are closely related). The major benefits and draw-backs associated with both avenues—as far as we are currently aware of—are highlighted. There are also various critical questions raised by nanosizing which ultimately deserve our attention.

In any case, to unlock the biological activity of active ingredients contained within plants and plant cells, it should be possible to nanosize a wide variety of such different plants and parts thereof (Figure 7). As long as they can be dried, do not contain excessive fats or oils, such natural plant materials should be suitable for simple milling and homogenization procedures. In fact, the activity of the particle preparations derived from the fruits of *S. incanum* and the bark of *P. erinaceus*, in both cases compares rather well with the ones observed for the respective extracts. This, in turn, may point to wider practical applications—not only of the nanosizing method, but also of the two plants involved in this study. Whilst *S. incanum* seems to be promising in a more agricultural context as a possible nematicide, *P. erinaceus* may possess antibacterial properties useful in the context of simple infections, for instance affecting the gastrointestinal tract or the skin. In both cases, toxic effects on human cells obviously need to be investigated in earnest. Yet as far as we can judge, neither agricultural nor topical applications seem to bear any excessive risks.

Besides the Jericho tomato and the African barwood, which have been used here simply to showcase the potential of the homogenization method, one may also envisage a wide range of additional plants commonly grown and highly abundant—often even as weed or waste—in developing countries with a rich flora, in particular in Africa, Asia and South America. Promising examples include, for instance, *Nauclea latifolia Sm.* or *Ocimum gratissimum* L., which already have some reputation as being effective against malaria and intestinal parasites. [9] Other sources of particular interest may well include various parasitic plants, which often also behave as weeds, and on one side are rich in biologically active ingredients and on the other tend to lack chlorophyll and other readily degrading substances. Indeed, there is plenty of choice as far as suitable plants are concerned.

Eventually, comprehensive studies on the nanosizing techniques, the particles obtained and their respective biological activities, physico–chemical and release properties will decide if such methods indeed provide a viable alternative to the extraction, isolation, formulation and delivery methods traditionally employed to move from a crude plant material to a practical application. As nanosizing “in one go” covers all these conventional methods from extraction to formulation (Figure 7), and since our initial results are certainly not negative, it is definitely worthwhile to give such methods and the resulting particles further consideration.

## 5. Conclusions

In essence, our study has lend support to the idea that nanosizing of plant materials may enable us to move from a crude, dried plant material to an applicable, “complete particle”-based delivery and release system in just one or a few simple steps. Whilst there are plenty of questions which remain to be addressed and answered in the future, none of these issues is insurmountable. Depending on the funds available, the methods for nanosizing can be varied and refined, ultimately resulting in more defined and stable particles. Release properties can also be controlled by such processes, and so can be the (physical) properties of the particle itself and the biological activity caused by the substances released from it.

Future studies will therefore not only focus on the preparation of a wide range of particles from an equally wide range of local plants, and of different, application-specific quality. They will also consider a much wider spectrum of possible applications in the fields of nutrition and cosmetics, in disease prevention and therapy. Here, cardiovascular, anti-inflammatory, anti-cancer and anti-infective agents may be at the forefront of investigation. Particles of *P. erinaceus*, for instance, have already shown some activity in this context and, after a more in-depth examination, could herald a new era for locally grown and produced particles against dysentery and simple skin infections.

Ultimately, a prime focus will also be on agricultural applications, as the amounts required in agriculture are considerably higher than in medicine, whilst the potential risk for humans is lower. *S. incanum* is one example for such possible agricultural applications, and for turning waste into value, but there are many more. “As long as it can be nanosized, it should be all right.”

## Abbreviations

The following abbreviations are used in this manuscript:

h	hours
HSS	High Speed Stirring
HPH	High Pressure Homogenization
LD	Laser Diffraction
PCS	Photon Correlation Spectroscopy
